# Cavernous Breast Hemangioma Mimicking an Invasive Lesion on Contrast-Enhanced MRI

**DOI:** 10.1155/2019/2327892

**Published:** 2019-04-09

**Authors:** Menelaos Zafrakas, Panayiota Papasozomenou, Panayiotis Eskitzis, Demetrios Zouzoulas, Glyceria Boulogianni, Thomas Zaramboukas

**Affiliations:** ^1^School of Health and Medical Care, Alexander Technological Educational Institute of Thessaloniki, Greece; ^2^1st Department of Obstetrics and Gynecology, Aristotle University of Thessaloniki, Greece; ^3^Technological Educational Institute of Western Macedonia, Ptolemais, Greece; ^4^Euromedica Private Diagnostic Center of Thessaloniki, Greece; ^5^Istodierevnitiki SA, Thessaloniki, Greece

## Abstract

Hemangiomas are vascular lesions, which are only rarely located in the breast. Larger breast hemangiomas may be detected by clinical examination, mammography, and breast ultrasound, whereas smaller lesions are usually incidental findings. We present a rare case of a 43-year-old woman with a cavernous hemangioma of the breast, presenting only on MRI and evading mammographic and ultrasonographic imaging. On breast MRI, a small lesion with irregular margins was detected in the right breast, and following gadolinium contrast medium administration, a type 3 curve, with rapid initial rise, followed by reduction in enhancement (washout) in the delayed phase was noted, raising suspicion for malignancy. The lesion could not be visualized on second-look targeted breast ultrasound and full-field digital mammography. A wide local excision was performed after 3 T MRI-guided hook wire localization and diagnosis of cavernous hemangioma was established histologically. Cavernous hemangioma is a rare breast lesion, with only few cases reported in the literature, and this is the first case with a presentation mimicking an invasive tumor on contrast-enhanced MRI.

## 1. Introduction

Vascular tumors of the breast are infrequent lesions and most cases are either angiosarcomas or hemangiomas [1, 2]. Cavernous hemangioma is considered to be the most common type of hemangioma found in the breast; however, only a few cases have been reported in the literature [3]. Cavernous hemangiomas are usually detected in other anatomic sites, including the liver, the brain, and the orbit [4–6]. Clinical diagnosis of mammary hemangioma is rather difficult, since most lesions are impalpable and most cases are coincidental microscopic findings after surgical procedures performed due to irrelevant indications [7, 8]. On the other hand, breast hemangiomas may be detectable by imaging tests, including mammography, breast ultrasound, and contrast-enhanced MRI. In the present paper, we report a rare case of cavernous hemangioma of the female breast presenting as a coincidental finding on contrast-enhanced MRI, mimicking an invasive tumor and evading mammographic and ultrasonographic detection.

## 2. Case Report

**Figure 1:**
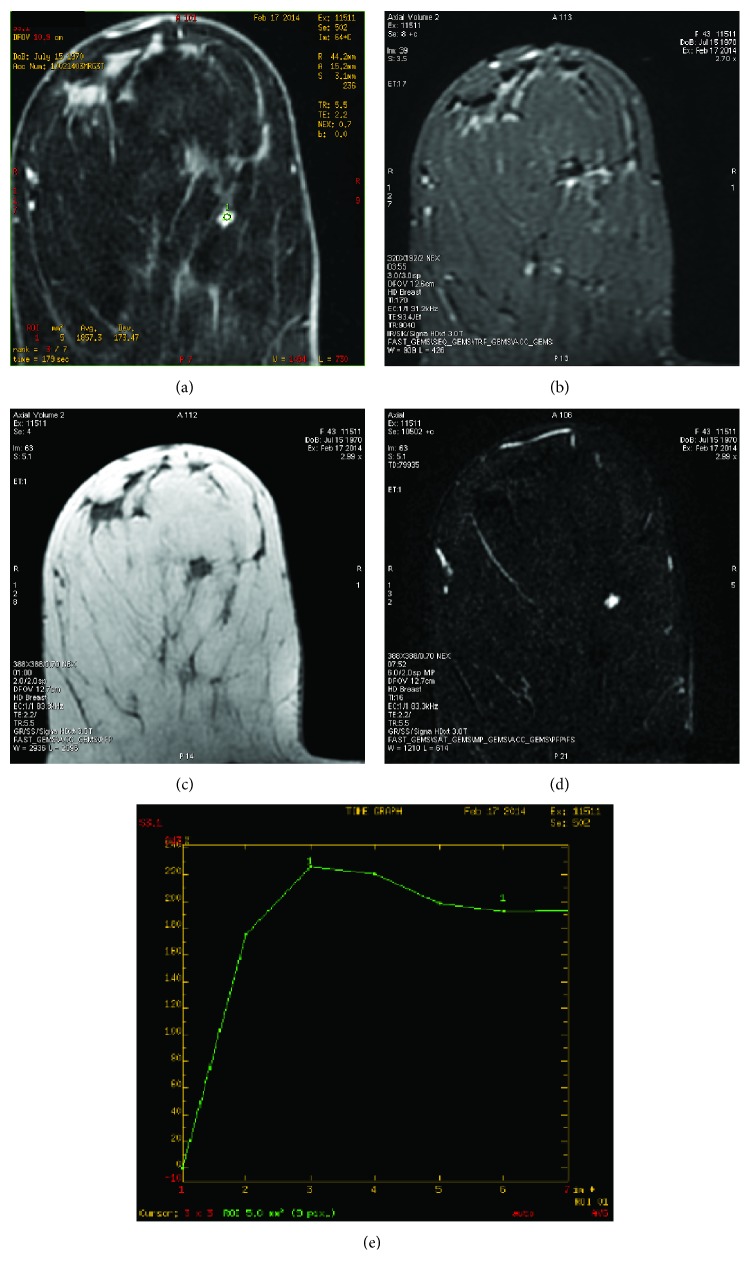
Contrast-enhanced MRI imaging of a cavernous hemangioma of the breast mimicking an invasive lesion. (a) Τ1W fat sat postcontrast image showing a small enhancing lesion measuring 5 mm, with irregular margins in the lower inner quadrant of the right breast. (b) STIR image showing the mass with low signal and central high signal. (c) Precontrast T1W non fat suppressed image showing an intermediate signal in the lesion. (d) Postcontrast T1W subtraction image: a small enhancing mass is seen. (e) A type III curve with rapid enhancement and delayed washout is observed.

**Figure 2:**
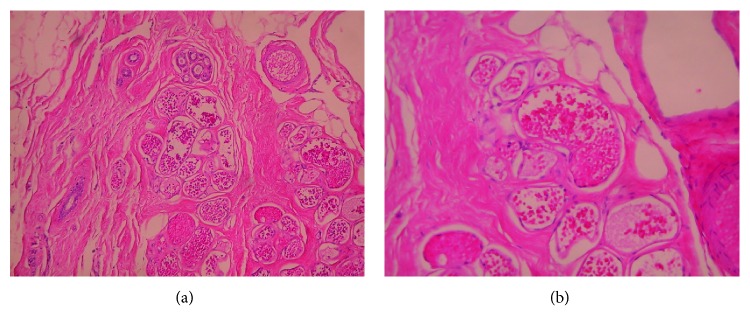
Microscopic views of a cavernous hemangioma of the breast mimicking an invasive lesion on contrast-enhanced MRI. (a) Cavernous breast hemangioma with thin-walled vascular spaces in contact with glandular breast tissue (Η+Ε ×100). (b) Cavernous breast hemangioma with thin-walled vascular spaces in contact with fatty tissue (Η+Ε ×200).

A 43-year-old woman, gravida 4, para 2, was referred due to a suspicious finding on 1.5 T contrast-enhanced MRI; the indication for MRI by the referring gynecologist was family history, with one close relative with breast cancer (her mother diagnosed at age 50). The patient's personal medical history was unremarkable. In particular, she had no prior history of breast disease or breast injury and she had not taken any exogenous hormones in the past; there was no palpable mass, skin changes, or axillary lymphadenopathy. Diagnostic full-field digital mammography and breast ultrasound were also unremarkable. However, on MRI, a small lesion with irregular margins measuring approximately 6 mm was detected in the right breast, in the lower inner quadrant. After gadolinium contrast medium administration, a type 3 curve, with rapid initial rise, followed by reduction in enhancement (washout) in the delayed phase was noted, raising suspicion for malignancy. In Figure 1, representative MRI views of the lesion are presented. The lesion could not be visualized on second-look targeted breast ultrasound and full-field digital mammography reevaluation. After thorough discussion with the patient and signed informed consent, a wide local excision was performed after 3 T MRI-guided hook wire localization. The suspicious lesion was excised with clear margins. Macroscopically, it was ovoid, soft, spongy, and dark red-brown with a maximal diameter of 5 mm. On microscopy, diagnosis of cavernous hemangioma was established; it consisted of dilated, congested hyperemic blood vessels, lined with endothelial cells; there were no signs of malignancy or atypia in the lesion and surrounding tissue. In Figure 2, representative microscopic views of the lesion are presented. Follow-up MRI two months later confirmed removal of the whole lesion. Today, almost five years later, the patient remains in good health without any signs of recurrence or any findings on imaging tests (annual mammography and ultrasound).

## 3. Discussion

Clinical diagnosis of mammary hemangioma is unusual, since most lesions are impalpable [9]; most of the times, they are incidental findings in specimens from procedures indicated for other causes, in particular in mastectomy (1.2%) and autopsy specimens (11%) [10]. Breast hemangiomas may occur at any age, even in childhood, with an age range between 18 months and 82 years [11]. Very rarely, hemangiomas may also present in association with breast implants [12] and in the male breast [13].

On mammography, a cavernous breast hemangioma may be visualized as a well-defined lobulated mass, with or without fine or coarse calcifications, due to scattered phleboliths [1, 11, 14]. On breast ultrasound, hemangiomas usually appear as hypoechoic, lobulated, well-circumscribed masses, lying parallel to the skin surface, although there have also been reports of isoechoic or mildly hyperechoic hemangiomas with poorly defined or microlobulated margins [9]. In other cases, however, breast hemangiomas, especially small lesions, may be not visible on mammography and/or breast ultrasound [15, 16]. In the present case, the lesion was a small cavernous hemangioma measuring 5 mm and as such it was not visible on mammography and breast ultrasound, even after targeted evaluation based on the MRI findings.

On MRI, breast hemangiomas typically appear as circumscribed masses of intermediate signal on T1-weighting and intermediate-to-high signal on T2-weighting [9]. In the present case, however, a cavernous hemangioma presented with irregular margins and a type 3 curve after contrast medium administration, mimicking an invasive lesion. A widely known limitation of MRI implementation in breast cancer screening is low specificity, leading to unnecessary biopsies and additional imaging [17, 18], especially if every enhancement is regarded as a potentially invasive lesion [19]. Some normal breast structures (vessels, nipples, and intramammary lymph nodes) could enhance and they should not be classified as suspicious findings [19]. Furthermore, it is noteworthy that non-mass-like enhancement is the major cause of false-positive breast MRI findings [17]. Given that breast hemangiomas are rare lesions, there are no guidelines regarding follow-up imaging for surveillance after excision; hence, follow-up imaging should be individualized on a case-by-case basis.

Differential diagnosis of cavernous hemangiomas of the female breast necessitates exclusion of the possibility of an angiosarcoma, especially if a lesion is palpable or symptomatic; such lesions should be regarded as malignant unless proven otherwise [2, 20, 21]. Whether a hemangioma may undergo malignant transformation to an angiosarcoma is controversial [3]. Core needle biopsy appears to be a reliable diagnostic tool for breast hemangiomas; however, due to the rarity of these lesions, histologic evaluation following core needle biopsy may be inconclusive, making wide local excision of these lesions the preferred treatment approach, also allowing definitive diagnosis [22].

In conclusion, despite its rarity, the possibility of a breast hemangioma should be considered in cases of incidentally detected suspicious lesions, especially in case of discordance between MRI and findings from other imaging studies. Furthermore, health care professionals taking care of asymptomatic women should be aware that screening MRI is indicated only in high-risk women after specialized risk assessment and that excessive use of this imaging modality may lead to unnecessary overdiagnosis and overtreatment.
